# Double Transgenic Pigs with Combined Expression of Human α1,2-Fucosyltransferase and α-Galactosidase Designed to Avoid Hyperacute Xenograft Rejection

**DOI:** 10.1007/s00005-014-0280-3

**Published:** 2014-02-20

**Authors:** Joanna Zeyland, Anna Woźniak, Barbara Gawrońska, Wojciech Juzwa, Jacek Jura, Agnieszka Nowak, Ryszard Słomski, Zdzisław Smorąg, Marlena Szalata, Urszula Mazurek, Daniel Lipiński

**Affiliations:** 1 Department of Biochemistry and Biotechnology, Poznan University of Life Sciences, Dojazd 11, 60-632 Poznan, Poland; 2 The Nano Bio Medical Centre, Poznan, Poland; 3 Institute of Human Genetics, Polish Academy of Sciences, Poznan, Poland; 4 Department of Biotechnology and Food Microbiology, Poznan University of Life Sciences, Poznan, Poland; 5 Department of Animal Reproduction, National Research Institute of Animal Production, Balice, Poland; 6 Department of Molecular Biology, Medical University of Silesia, Sosnowiec, Poland

**Keywords:** α1,2-fucosyltransferase, α-galactosidase, Hyperacute xenograft rejection, Xenotransplantation

## Abstract

Hyperacute rejection (HAR) depends on the response of xenoreactive antibodies principally against porcine α-Gal epitope. Methods eliminating HAR include *GGTA1* inactivation, regulation of the complement system and modification of the oligosaccharide structure of surface proteins in donor’s cells. Transgenic animals designed for the purpose of xenotransplantation with single modification do not display full reduction of the α-Gal epitope level, which means that a accumulation of several modifications in one transgenic individual is needed. The aim of the study was to create a molecular and cytogenetic profile of a double transgenic animal with α1,2-fucosyltransferase and α-galactosidase expression. As a result of interbreeding of an individual with α1,2-fucosyltransferase expression with an individual with α-galactosidase expression 12 living piglets were obtained. PCR revealed the pCMVFUT gene construct was present in four individuals and pGAL-GFPBsd in three, including one with a confirmed integration of both the gene constructs. Fluorescence in situ hybridization confirmed the site of transgene integration, which corresponded to the mapping site of the transgenes which occurred in the parental generations. Karyotype analysis did not show any changes in the structure or the number of chromosomes (2*n* = 38, XX). As for the results pertaining to the single transgenic individuals, expression analysis demonstrated a high extent of α-Gal epitope level reduction on the surface of cells, whereas human serum cytotoxicity tests revealed the smallest decrease in longevity of cells in the obtained double transgenic individual (4.35 %). The tests suggest that the co-expression of both the transgenes leads to a considerable reduction of the α-Gal antigen level on the surface of cells and a decrease of xenotransplant immunogenicity.

## Introduction

The use of tissues and organs of transgenic animals is a subject of state-of-the-art and innovative biotechnological research, focused primarily on biomedicine. Slow increase in the number of organ donors and a growing number of patients who need a transplant is a very serious medical problem. This makes researchers look for alternative ways of treatment, including interspecies transplantations.

Given similar anatomical and physiological parameters of humans and pigs, the latter seem to be the most adequate animal species for interspecies transplantation in the animal-to-human model. Considerable phylogenetic distance may limit the prevalence of infections with zoonotic pathogens, especially when animal breeding is strictly controlled. However, the distance alone is a source of serious immunological barriers. Due to this fact, the success of xenotransplantation depends on the elimination of the xenoreactive antibody response against the porcine α-Gal epitope. This antigen is present in glycolipids and glycoproteins of the endothelial cells surface in all mammalian organisms with the exception of human and higher primates. Its synthesis is catalyzed by transmembrane protein called α1,3-galactosyltransferase (α1,3GT, EC2.4.1.151) and results from the adding of galactose residue to *N*-acetyllactosamine. This results in the already mentioned immunological barrier in the pig-to-human combination because of the activity of antibodies recognizing the α-Gal antigen. Anti-Gal antibodies are responsible for hyperacute rejection (HAR), which refers to cell lysis induced by the complement system activity and destruction of cells by macrophages and NK cells as a result of antibody-dependent cellular cytotoxicity (Suchanowska and Czerwiński 2009). It is estimated that approximately 1 % of antibodies in the human organism is of anti-Gal nature and the induction of their formation results from the immunization with α-Gal antibodies derived from the gastrointestinal bacteria. Anti-Gal antibodies of isotype IgG comprise the most numerous group, whereas IgM immunoglobulins are responsible for the strongest reaction (McMorrow et al. 1997; Sandrin et al. 1993).

The key factor of preventing the rejection of xenografts is the development of methods of controlling or limiting the hyperacute reaction of the immunological system through decreasing the amount of the α-Gal epitope on the surface of donor cells. One of the ways of obtaining pigs that would comprise a source of organs for xenotransplantation by means of eliminating α-Gal epitope is α1,3-galactosyltransferase inactivation (GTKO). Heart transplant from a α1,3GT knock-out pig prolonged the survival of the xenotransplant in the baboon organism to 6 months, whereas in the case of kidney transplant it was 3 months (Cozzi et al. 2000; Kuwaki et al. 2005; Tseng et al. 2005; Yamada et al. 2005). However, approximately 2 % of the initial amount of the α-Gal antigen remains on the surface of cells in transgenic GGTA1 knock-out pigs. Thus, the simultaneous use of several strategies for decreasing the amount of the xenogeneic epitope is justified.

An alternative method of decreasing the amount of such epitope and mitigating the HAR is to introduce a gene coding for human α1,2-fucosyltransferase (H, HT transferase) into the porcine genome. This enzyme catalyzes the addition of fucose using the same receptor (*N*-acetyllactosamine) as α1,3-galactosyltransferase. The activity of the two enzymes is competitive (Larsen et al. 1990; Sandrin et al. 1995). Pigs carry an endogenous gene coding for α1,2-fucosyltransferase; however, the H epitope produced by this enzyme is not expressed on the endothelial surface of blood vessels (Oriol et al. 1993; Sharma et al. 1996). Expression of α1,2-fucosyltransferase does not, however, allow for a complete elimination of the α-Gal epitope from the surface of cells. Thus, a co-expression of α1,2-fucosyltransferase and α-galactosidase is suggested, as it removes terminal residues of epitope, d-galactose from the surface of pig cells by means of digestion (Luo et al. 1999).

The aim of the study was to prepare a molecular and cytogenetic profile of a double transgenic animal with α1,2-fucosyltransferase and α-galactosidase expression designed to avoid HAR to assess the effectiveness of both the enzymes in one organism and compare it with the results obtained for organisms with a single modification (with either α1,2-fucosyltransferase or α-galactosidase). According to Cooper (2012) article and our knowledge, this is the first official report of double transgenic pigs with combined expression of α1,2-fucosyltransferase and α-galactosidase.

## Materials and Methods

### Animal Samples Collection

Ear biopsy specimens were collected from animals born as a result of a interbreeding of heterozygous individual TG4337 (F1 generation) derived from a founder TG1154 with introduced α1,2-fucosyltransferase gene, and heterozygous individual TG284 from the F1 generation, descending from a founder TG252 (F0) with introduced α-galactosidase gene (Lipiński et al. 2010; Zeyland et al. 2013). The process of obtaining transgenic animals from F0 generations (founders) was previously described in the above mentioned works.

### Screening of Transgenes

Analysis included genomic DNA isolation from biopsies of all 12, potentially double transgenic animals with the use of proteinase K. PCR reaction was conducted on DNA template. Two pairs of primers were used for amplification for each transgene. For screening of pGAL-GFPBsd both F primers were complementary to the hEF-1α promoter sequence, and both R primers were complementary to the sequence of α-galactosidase. PCR product of 464 bp was formed with the use of the first pair of F1 (5′-GGGGAGGGGTTTTATGCGATGGAG-3′) and R1 (5′-CTGGCTCTTCCTGGCAGTCA-3′) primers, whereas the second product of 861 bp with the use of F2 (5′-ACCAGTTGCGTGAGCGGAAAGATG-3′) and R2 (5′-GGCGAATCCCATGAGGAAAG-3′) primers. For screening of pCMVFUT forward primers were located in the cytomegalovirus promoter region and reverse primers in the region coding of the α1,2-fucosyltransferase. PCR product of 144 bp was amplified with F3 (5′-GCAGAGCTGGTTTAGTGAA-3′) and R3 (5′-ATATGGAGGAAGAAGATTACAGAG-3′) primers, whereas the 343-bp fragment was obtained with F4 (5′-ATGGTGATGCGGTTTTG-3′) and R3 (5′-ATATGGAGGAAGAAGATTACAGAG-3′) primers. PCR reaction was conducted in 20 μl in the following conditions: 94 °C, 40 s; 60 °C, 30 s; 72 °C, 60 s; 35 cycles. The reaction contained 100 ng of genomic DNA, 10 pM of primers, 7.5 nM of dNTP, 1 × buffer, 1U of DNA *Taq* polymerase (Sigma Aldrich, USA).

### Skin Fibroblast Isolation and Cultivation

Primary fibroblast cell lines were started from ear biopsy specimens of pigs. Ear biopsy specimens collected in sterile conditions were placed in a solution containing antibiotics (50 μg/ml of gentamicin sulfate, 100 IU of penicillin and 50 μg/ml of streptomycin). The ear biopsy specimens were cut and then digested in 5 ml tripsin-EDTA solution (0.25 % tripsin, 0.02 % EDTA). The cell sediment was suspended in enriched Dulbecco’s modified eagle medium [DMEM; 20 % fetal bovine serum (FBS), 1 % antibiotic/antimycotic] and cultivated at the temperature of 37 °C at 5 % CO_2_ content in a humid environment. The culture was established in sterile conditions, using a cabinet with laminar air flow (Heraeus, Germany). The medium was replaced every 3 days. Cultures of 80 % confluence were passaged.

### Obtaining Metaphase Preparations

Colcemid (0.05 μg/ml) was added to culture dishes of 80 % confluence. The cultures were incubated at 37 °C for 2 h to stop cell divisions in the mitotic metaphase by means of damaging the spindle apparatus. Next, the cells were subjected to osmotic shock by adding 0.4 % KCl solution. After 30-min incubation, the material was fixed by adding a cold mixture of methanol and acetic acid (3:1) three times. The number and dispersion of metaphase plates was assessed by determining a mitotic index during the observation through a Nikon Eclipse E400 optical microscope with a phase contrast objective.

### Transgene Mapping

Molecular probes (pCMVFUT and pGAL-GFPBsd) were directly labeled with FITC in random priming. Next, fluorescence in situ hybridization (FISH) was conducted. The obtained chromosome preparations were digested with RNase (final concentration of 100 μg/ml in a 2 × SSC solution), pepsin (final concentration of 100 μg/ml in 0.01 N HCl), washed [2 × SSC, phosphate buffered saline (PBS) plus MgCl_2_] and dehydrated in alcohols in order of their increasing strength. Then, co-denaturation on a heating panel was carried out at 80 °C for 90 s. The analysis of the hybridization signal was conducted by means of a Zeiss Axiophot fluorescent microscope connected with a CCD camera. The obtained images were analyzed and archived by means of MetaSystems 2004, ISIS Version 5.0. in UV light, using a set of DAPI/FITC/Texas Red/Triple color filters and an immersion objective (100 ×).

Karyotype evaluation in the investigated animals was based on the obtained GTC band pattern and available patterns of porcine karyotypes (Gustavsson 1988). The previously collected image documentation concerning GTC band staining in the investigated animals was processed using the MetaSystems 2004, ISIS Version 5.0. software to arrange the chromosomes in homologous pairs and chromosome groups corresponding to the pattern.

### Southern Hybridization Analysis

For Southern analysis total genomic DNA from wild-type and analyzed transgenic pigs was extracted from white blood cells from peripheral blood with the method based on guanidine isothiocyanate. Each DNA sample (8.3 μg) was digested with restriction enzyme *Eco*RI and hybridized with a probe of 464 bp for pGAL-GFPBsd. For pCMVFUT, DNA was digested with restriction enzyme *Bam*HI and hybridized with a probe of 144 bp, respectively. The probe was a 32P-dATP-labeled PCR product of 464 bp formed on plasmid DNA (pGAL-GFPBsd) with the use of the first pair of primers (F1 and R1) used for integration analysis and (described above). For pCMVFUT the probe was a 32P-dATP-labeled PCR product of 144 bp formed on plasmid DNA with the use of the pair of primers (F3 and R3) used for integration analysis and (described above). Both enzymes digest the genomic DNA of pig as well as plasmid sequence (only one site). Digestion of plasmid sequence with *Eco*RI gives a fragment of 7.3 kb, and digestion with *Bam*HI gives a fragment of 6.5 kb which are identical with a size of the introduced genetic constructs. Additionally, for determining transgene copy number, to the digested DNA of wild-type pig, known amounts of plasmid containing transgene insert DNA was added. Then, DNA samples were electrophoresed through 1 % agarose gel in 1 × TBE, 40 V, overnight. Denatured and neutralized DNA immobilized in dried gel was hybridized to a transgene specific probe, overnight (55 °C, 5 × SSPE, 0.1 % SDS).

### Determination of the Absolute Number of Transgene Copies in Transgenic Pigs

Since 1 ng of DNA corresponds to the molecular mass of 606,060,600 MDa and the molecular mass of the swine genome amounts to 1,820,000 MDa, 1 ng of swine genomic DNA contains approximately 333 copies of the genome. 8.3 μg of swine genomic DNA sample per gel line were used; hence each of the examined samples contained about 2,763,900 copies of the swine genomic DNA. In order to calculate the number of copies of the transgene incorporated into a single genome of transgenic animals, the absolute number of copies of the transgene in the samples of known transgene copy number (22,000,000 copies of pGAL-GFPBsd and 8,875,000 copies of pCMVFut, respectively) was divided by the number of copies of the genomic DNA. In the case of heterozygotic transgenic animals, the transgene occurs only on one of the pair of homologue chromosomes; therefore, to calculate the number of transgene copies built into one of the chromosomes the obtained results of calculations was multiplied by 2.

### Flow Cytometry

Having reached full confluence, the skin fibroblasts cultivated in vitro were washed with Hanks solution and tripsinized (0.25 % trypsin, 0.02 % EDTA). Fibroblasts were collected by centrifugation. The cells were suspended and incubated in PBS containing lectin (10 μg/ml) and 5 % FBS, at 4 °C, for 45 min. Lectin BS-IB4 isolated from *Griffonia simplicifolia* (*Bandeiraea simplicifolia*) conjugated with AlexaFluor 647 fluorochrome (Invitrogen) was used for specific detection of the α-Gal epitope. Flow cytometer (cell sorter) BD FACS Aria™III (Becton–Dickinson, USA) with four lasers (375, 405, 488 and 633 nm), eleven detectors of fluorescence and dispersed laser light detectors—forward scatter and side scatter were used for the analysis of α-Gal residue on the surface of examined cells. Optical alignment and functional stability tests were conducted with cytometer setup and tracking system (Becton–Dickinson, USA). FACSFlow (Becton–Dickinson, USA) was used as sheath fluid. The following cytometer configuration was applied: 85 μm cell sorting nozzle and sheath fluid pressure of 45 psi. Characteristics of cells were obtained based on two non-fluorescent parameters: forward scatter and side scatter and one fluorescent parameter: red fluorescence (FL1 detector) from the AlexaFluor 647 fluorochrome—detection with the use of a 660/20 optical filter. Red laser (633 nm) was used for AlexaFluor 647 fluorochrome excitation. Cytometric analysis was conducted based on specific detector settings, which convert the collected signals into a logarithmical scale. Forward scatter detector was equipped with an optical filter of neutral density to weaken the dispersed light reaching the detector and enable converting the population of analyzed cells on two-dimensional scale in a graph showing relations between forward scatter and side scatter. The threshold was set at the signal of forward scatter detector. Data presented in the logarithmical scale divided into four decades in the form of signal surface from different detectors (Forward Scatter-A, Side Scatter-A and FL1-A) and analyzed with the use of FACS DIVA software (Becton–Dickinson, USA). The analysis of fluorescence signals preceded doublets discrimination procedure with the use of height versus width scatter signals measurement, to discriminate single events (cells) from conglomerates. The populations were then defined by gating in the dot plots of red fluorescence (FL1) versus side scatter signals. Each sample was analyzed four times. Determining the levels of α-Gal epitope on the surface of the analyzed cells was conducted based on the median values of fluorescence signals from FL1 detector in populations defined on a two-dimensional graph (FL1 and side scatter).

### Human Serum Cytotoxicity Test

Having reached full confluence, the skin fibroblasts cultivated in vitro were rinsed with Hanks solution and trypsinized. The fibroblasts were collected by centrifugation and the debris was washed with Hanks solution and suspended in 200 μl of one of three mediums: 50 % DMEM and 15 % FBS in PBS—basic medium; 50 % human serum (HS) in PBS—test medium; 50 % thermally inactivated HS in PBS—control medium. The cells were incubated at 37 °C for 30 min. Then, 20 μl of 0.4 % trypan blue was added to 20 μl of cell suspension and incubated for 5 min at room temperature. Living and dead cells were counted in Bürker’s chamber of 0.9 mm^3^. Cells that were over or touching the top and left line of each square of 1 mm^2^ were counted while the cells that were over or touching the bottom or right line were ignored. Each analysis was repeated 12 times. The percentage of living cells was calculated for each repetition. Then average numbers of living cells, dead cells and all cells from 12 repetitions were calculated.

## Results

### Obtaining Transgenic Pigs with α-Galactosidase and α-1.3 Fucosyltransferase

**Fig. 1:**
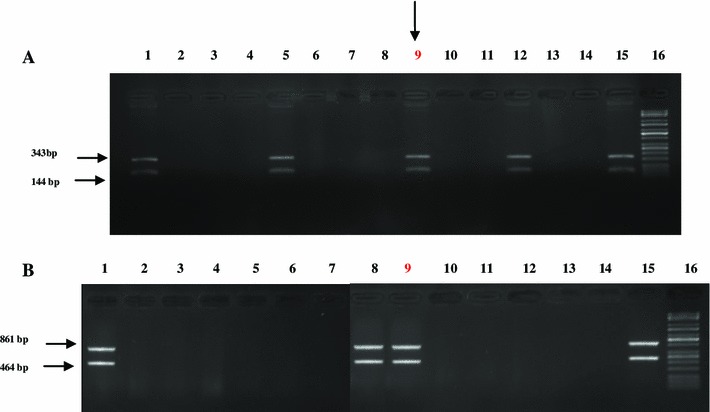
Screening of pCMVFUT and pGAL-GFPBsd gene construct. PCR was used to amplify DNA fragments of 464 and 861 bp for pGAL-GFPBsd and 144 and 343 bp for pCMVFUT. Separation of DNA fragments was conducted in 1.5 % agarose gel. **a** Analysis of pCMVFUT integration with genomic DNA of 12 piglets. *Lanes 1, 5, 9, 12*: DNA from piglets TG588, TG604, TG632, TG605, respectively, *lanes 2*–*4, 6*–*8, 10, 11*: DNA from piglets analyzed as nontransgenic individuals; *lane 13*: negative control (wild-type pig DNA); *lane 14*: negative control (without DNA); *lane 15*: positive control (pCMVFUT gene construct); *lane 16*: size marker (DNA KAPA Universal Ladder). *Red nine* indicates double transgenic piglet TG632. **b** Analysis of pGAL-GFPBsd integration with genomic DNA of 12 piglets. *Lanes 1, 8, 9*: DNA from piglets TG628, TG629, TG632, respectively; *lanes 2*–*7, 10*–*12*: DNA from piglets analyzed as nontransgenic individuals; *lane 13*: negative control (wild-type pig DNA); *lane 14*: negative control (without DNA); *lane 15*: positive control (pGAL-GFPBsd gene construct); *lane 16*: size marker (Kappa size marker). *Red nine* indicates double transgenic piglet TG632

As a result of interbreeding of TG284 with TG4337, 12 living piglets (6 sows and 6 boars) were obtained. The piglets were subjected to integration analysis with regard to the presence of both the transgenes by means of PCR. The obtained animals included four transgenic individuals with confirmed integration of the pCMVFUT gene construct and three transgenic individuals with confirmed integration of the pGAL-GFPBsd gene construct (including one with an already confirmed integration of pCMVFUT transgene). Last six analyzed pigs were nontransgenic individuals (Fig. 1). Most further analyses were conducted only for a double transgenic animal, i.e. TG632 sow, or double transgenic animal and representatives of monotransgenic animals: one with confirmed integration of the pCMVFUT gene construct (TG588) and one with confirmed integration of the pGAL-GFPBsd gene construct (TG628).

### Cytogenetic Analysis

**Fig. 2:**
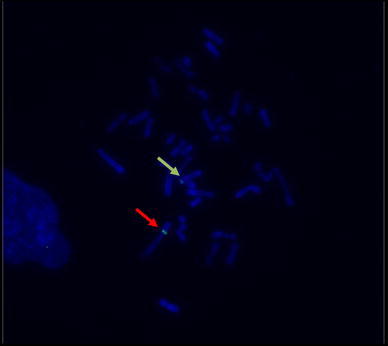
Post-hybridization image of metaphase chromosomes from interphase nucleus of cell line derived from skin fibroblasts of TG632. Molecular probe complementary to plasmid pGAL-GFPBsd (probe labeled with FITC—*color green*), and plasmid pCMVFUT (probe labeled with FITC—*green*). Chromosomes stained with DAPI—*blue*. Heterozygous configuration of transgenes—signal on chromosome 11p12 (pGAL-GFPBsd—*red arrow*) and chromosome 14q28 (Pcmvfut—*yellow arrow*)

**Fig. 3:**
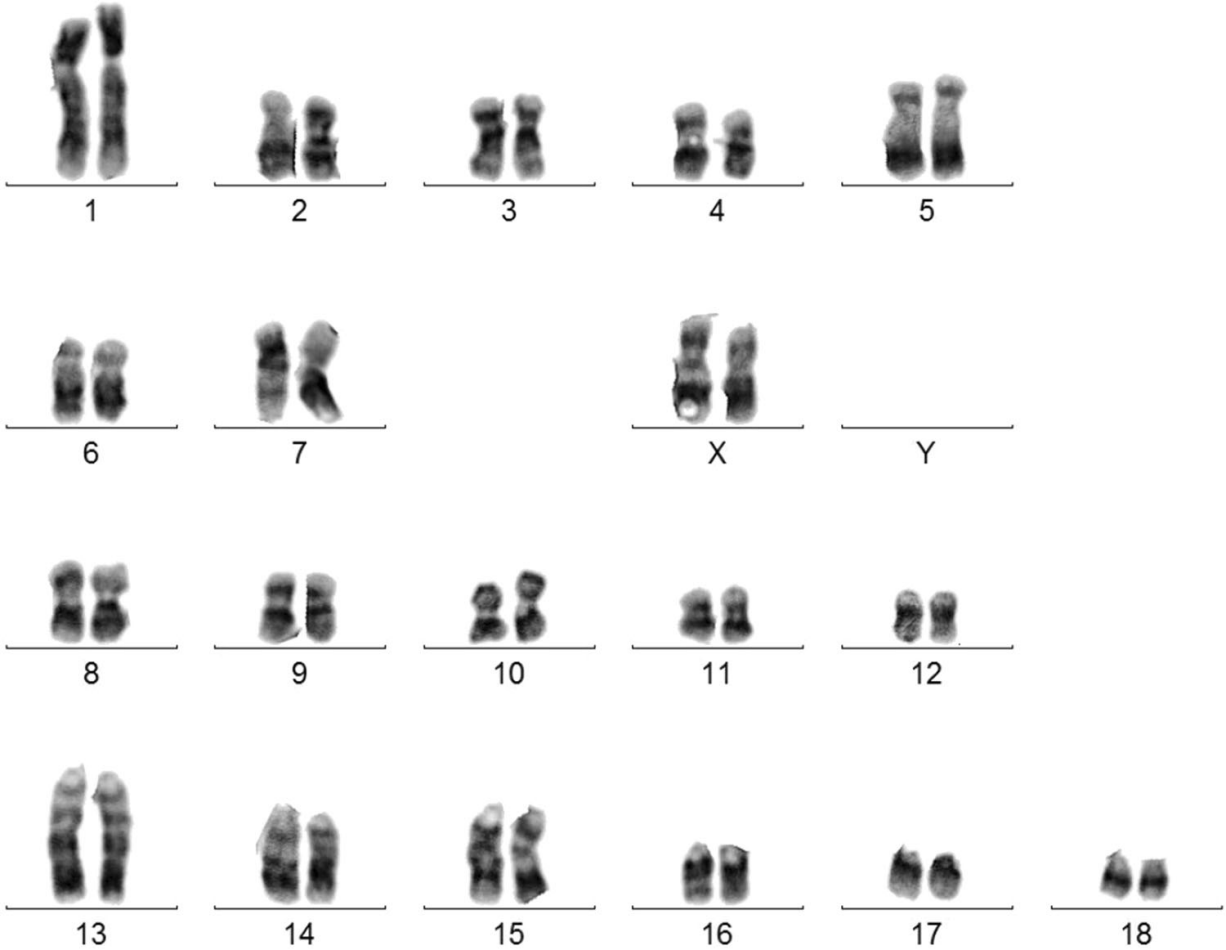
Correct karyotype of pig TG632 (2*n* = 38, XX)

FISH was conducted with the use of molecular probes complementary to the pCMVFUT (first) and pGAL-GFPBsd (second) plasmids directly labeled with the FITC fluorochrome (green). Metaphase chromosomes were stained by DAPI (blue). In transgenic sow TG632, both transgenes were detected in a heterozygous arrangement, pGAL-GFPBsd on chromosome 1p12 and the pCMVFUT transgene in the q28 region of chromosome 14, which corresponds to the localizations of the previously mentioned trangenes in the F0 transgenic individuals, founders TG1154 and TG252 (Fig. 2). Karyotype evaluation in transgenic individuals was carried out by staining the digested chromosomes with Giemsa stain. The obtained band pattern of chromosomes was compared with the standard karyotype of the domestic pig. No changes were observed in the structure or number of chromosomes (2*n* = 38, XX; Fig. 3).

### Southern Hybridization Analysis and Transgene Copy Number

**Fig. 4:**
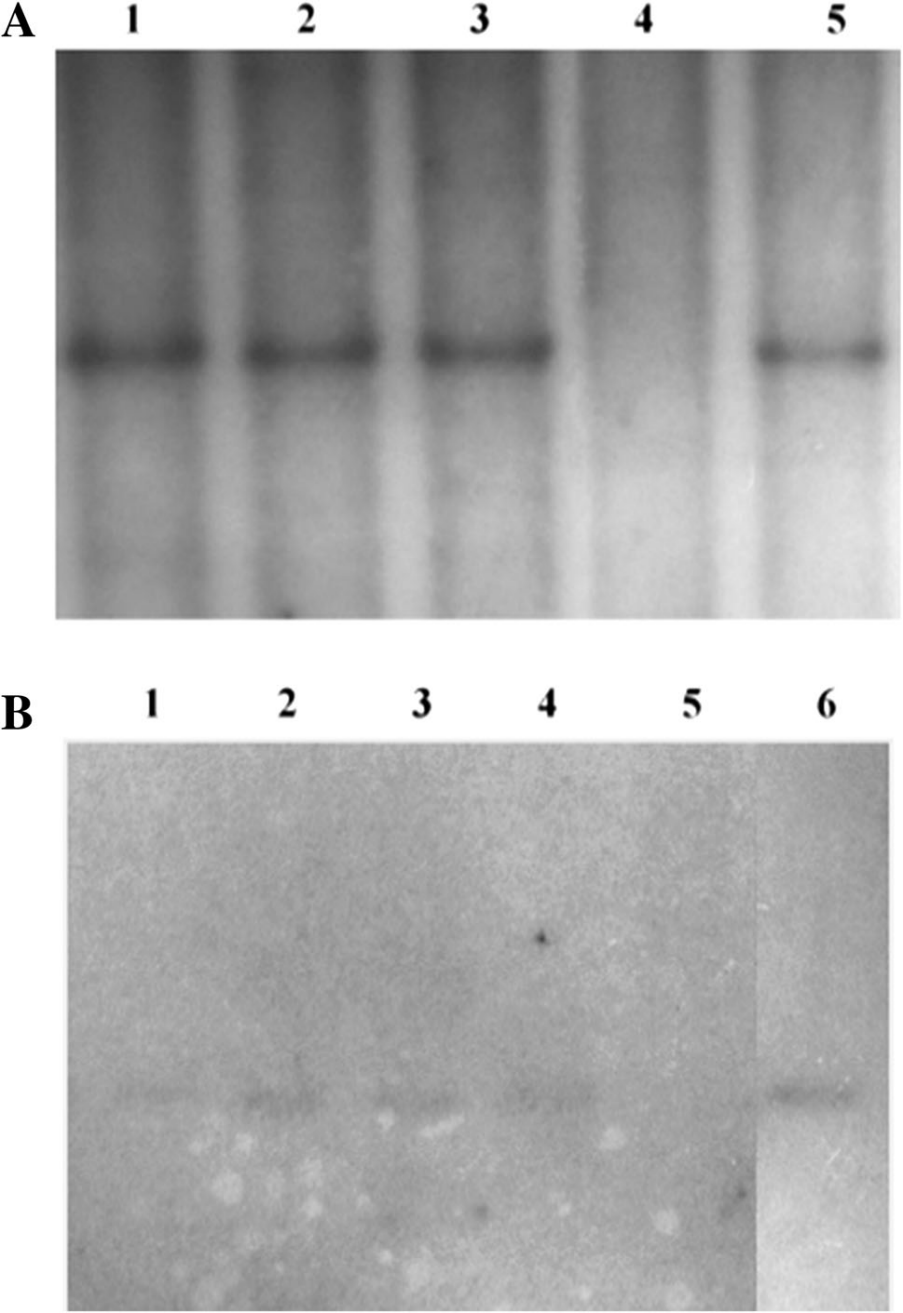
Confirmation of the presence of pCMVFUT and pGAL-GFPBsd gene construct in transgenic pigs by Southern hybridization analysis. **a** DNA from three transgenic piglets and wild-type pig, digested with *Eco*RI, fractioned on an agarose gel and hybridized with transgen-specific probe (464 bp for pGAL-GFPBsd). *Lanes 1*–*3*: DNA from piglets TG628, TG629 and TG632, respectively; *lane 4*: control DNA (wild-type pig); *lane 5*: transgene fragment mixed with nontransgenic genomic DNA representing 16 copies. **b** DNA from four transgenic piglets and wild-type pig, digested with *Bam*HI, fractioned on an agarose gel and hybridized with transgen-specific probe (144 bp for pCMVFUT). *Lanes 1*–*4*: DNA from piglets TG588, TG604, TG605 and TG632, respectively; *lane 5*: control DNA (wild-type pig); *lane 6*: transgene fragment mixed with nontransgenic genomic DNA representing four copies. Note that both probes gave positive signal in case of DNA from TG632 what indicates that piglet as double transgenic animal

To confirm integration of both transgenes, we conducted Southern analysis, which confirmed the presence of pCMVFUT transgene in TG588, TG604, TG605 and TG632 and presence of pGAL-GFPBsd in TG628, TG629 and TG632 (Fig. 4). By using known concentrations of the transgene mixed with wild-type DNA, we were able to estimate expected copy number in each of the animals. We did not use the absolute number of copies of the transgene in titration samples to calculate the number of copies of the transgenes incorporated into a single genome, because it was done before for single transgenic animals (founders) (Lipiński et al. 2010; Zeyland et al. 2013). In the current analysis, the approximate number of pGAL-GFPBsd transgene copies integrated was estimated as 16 and the approximate number of pCMVFUT transgene copies integrated was estimated as 3. The obtained results are consistent with the results of our previous experiments.

### Flow Cytometry

**Fig. 5:**
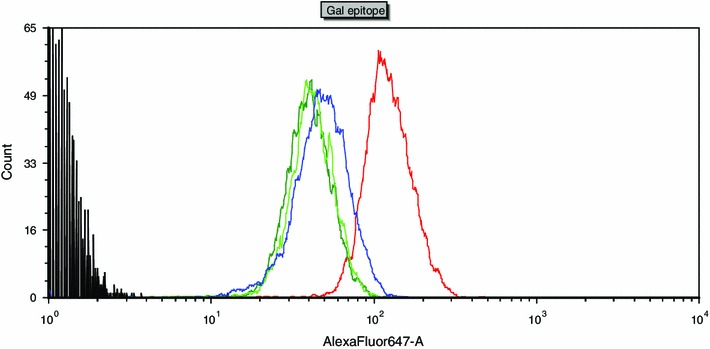
Transgene expression analysis by flow cytometry. Fibroblast derived from the following animals were analyzed: non-transgenic pig, TG588 transgenic pig expressing α1,2-fucosyltransferase, TG628 transgenic pig expressing α-galactosidase and TG632 transgenic pig expressing both α1,2-fucosyltransferase and α-galactosidase labelled by BS-IB4 lectin (detects α-Gal epitope), conjugated with AlexaFluor 647. *X axis* shows the fluorescence intensity, *Y axis* shows the number of cells, which were not labelled (*black line*), labelled non-transgenic cells (*red line*), cells from transgenic animal TG588 (*blue line*), cells from transgenic animal TG628 (*dark*-*green line*) and cells from transgenic animal TG632 (*light*-*green line*). The analysis showed decreased level of α-Gal antigen in transgenic animals compared to non-transgenic animals. The fluorescence fell by 60 % for the pig with α1,2-fucosyltransferase (TG588), 58.9 % for the pig with α-galactosidase expression (TG628) and 66.9 % for the pig with both α1,2-fucosyltransferase and α-galactosidase (TG632)

The flow cytometry method was used to assess how the presence of transgenes affects the α-Gal epitope level. The analysis was conducted on fibroblast lines derived from a non-transgenic animals which subjected to PCR displayed only a transgene coding for α1,2-fucosyltransferase (TG588) or only a transgene coding for α-galactosidase (TG628) and from individual TG632 with α1,2-fucosyltransferase and α-galactosidase. Before performing the analysis of enzyme functionality at the protein level by measuring the amount of the α-Gal antigene on the cell surface by flow citometry, the enzyme was labeled by BS-IB4 lectine conjugated with AlexaFluor 647 fluorochorme. BS-IB4 lectine isolated from *G. simplicifolia* (*B. simplicifolia*) specifically binds the α-Gal epitope and the fluorescent intensity of AlexaFluor 647 allows for determination of the relative amount of labelled antigens on the surface of the examined cells. Median values of fluorescence signals from FL1 detector (AlexaFluor 647) were proportional to α-Gal epitope levels on the surface of the analyzed cells. Staining with BS-IB4 lectine showed a significant reduction of fluorescent intensity in the case of fibroblasts from both the monotransgenic animals and the TG632 double-transgenic sow. The individuals with the pCMVFUT transgene (TG588), pGAL-GFPBsd transgene (TG628) and α1,2-fucosyltransferase and α-galactosidase expression (TG632) displayed the following decrease in the α-Gal epitope expression: 60, 58.9 and 66.9 % (triple reduction of the epitope level), respectively (Fig. 5).

### Human Serum Cytotoxicity Test

Another test verifying transgene functionality was the survival rate analysis of the skin fibroblasts derived from the two monotransgenic pigs and one double-transgenic pig TG632 as compared with the survival rate of other wild-type cell lines in the presence of human serum acting as a source of complement system elements. Activation of the complement system results in a number of reactions which result in the formation of a complex attacking the membrane. It is responsible for forming channels in the cellular membrane that deform its structures through changing lipid orientation and releasing liposomes. As a result of the activity of membrane-attacking complex, destruction of the recognized cells takes place. Control medium contained 50 % of inactivated human serum where the components of human complement system should not be active. This medium was used as toxicity control of compounds not belonging to the complement system, which were present in the human serum. Survival rate analysis showed that there is no statistically significant difference between the survival rate of cells in basic medium (50 % DMEM and 15 % FBS in PBS) and in control medium (50 % thermally inactivated HS in PBS), so it is not the source of toxic compounds. The survival rate was determined by incubation of cell suspension with a solution of trypan blue, a dye which penetrates the structure of dead cells whose membrane is fragmented.

**Fig. 6:**
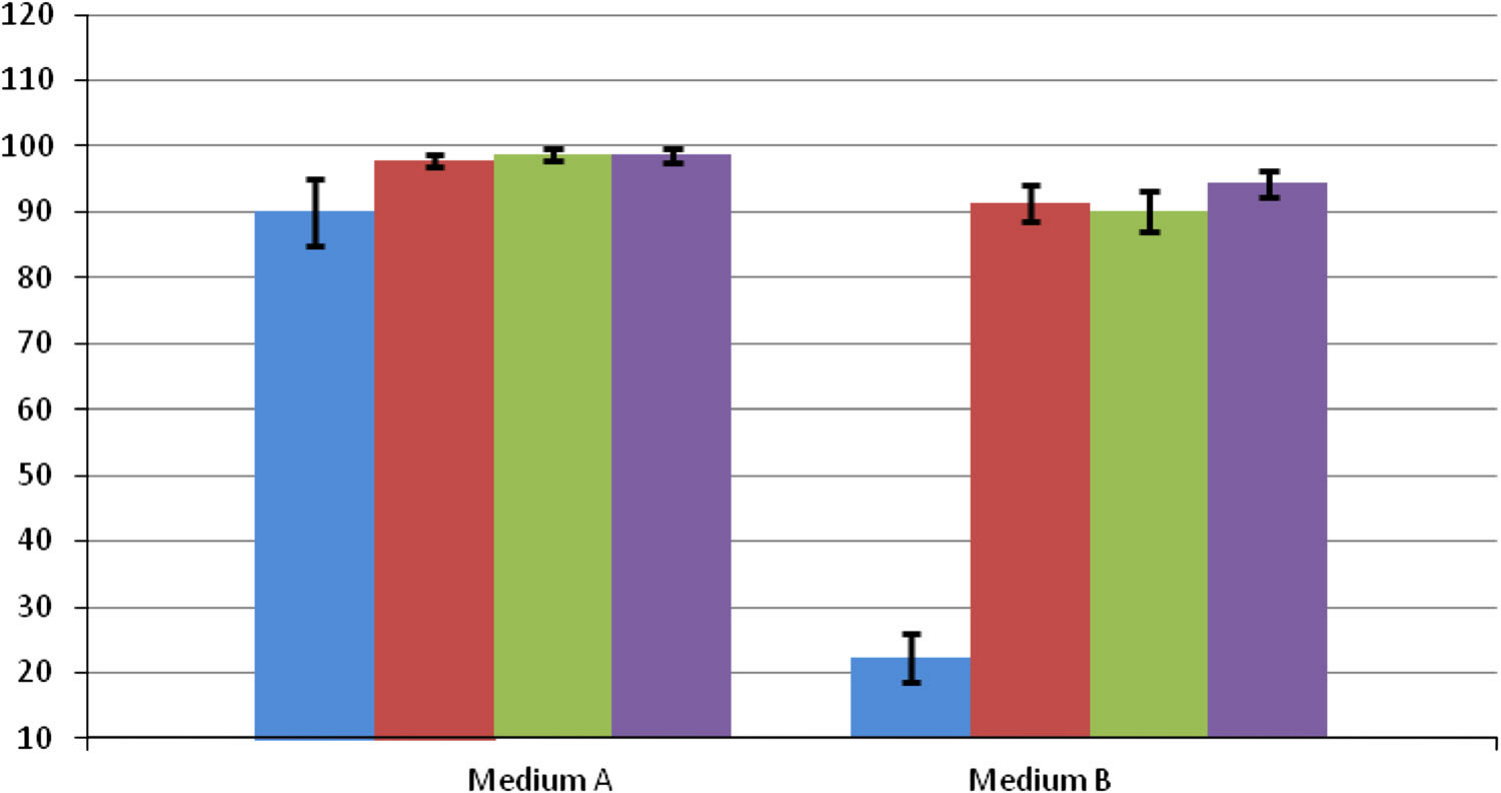
Survival rate analysis with standard deviations for cells from wild-type animal (*blue*), from TG588 expressing only α1,2-fucosyltransferase (*red*), TG628 animal with α-galactosidase expression (*green*) and TG632 with both α1,2-fucosyltransferase and α-galactosidase expression (*purple*) measured in the basic medium (A) and in test medium (B) containing 50 % of human serum. The survival rate was measured by an average number of living cells compared to the total number of counted cells in 12 repetitions. The percentage cell survival rate was marked on *Y axis*

The analyses conducted for test medium, where living cells constituted only 22.33 % (± 3.66 %), showed that the compounds present in the serum affected adversely the survival rate of wild-type pigs compared to basic medium (90.01 %, ± 5.11 %) with no elements of the human complement system. Similar comparative analysis conducted for a line of fibroblasts derived from a monotransgenic animal (TG628) expressing α-galactosidase revealed a minor decrease of cell survival rate in test medium (90.15 %, ± 3.06 %) in relation to the basic medium (98.77 %, ± 0.90 %). Similar survival rate analysis was conducted for a cell line derived from transgenic animal TG588 expressing α1,2-fucosyltransferase. The presence of transgene protected the skin fibroblasts from the complement system activity and the percentage loss of living cells was not significant. The survival rate measured by an average number of living cells compared to the total number of counted cells in 12 repetitions amounted to 97.88 % (± 1.21 %) in the basic medium and 91.31 % (± 2.69 %) in the test medium. The survival rate measured by an average number of living cells compared to the total number of counted cells in 12 repetitions amounted to 98.67 % (± 1.03 %) in the basic medium and 94.32 % (± 2.11 %) in the test medium for double transgenic cells derived from TG632 (Fig. 6).

## Discussion

HAR is the basic immunological barrier that makes transplanting pig organs to humans unsuccessful. There are three main directions that can be followed to overcome this barrier: depletion of anti-pig antibodies by the development of homozygous pigs for α1,3-galactosyltransferase gene-knockout, enzymatic removal of Galα1,3-Gal epitope or complement inhibition, or some combination of these approaches (Pierson 2009). When research on xenotransplantation was in its beginnings, there were several concerns about pigs with GTKO phenotype. First, possible indispensability of α-Gal in pigs and its essentiality for cellular function; second, the presence of another galactosyltransferase, which is capable of producing the α-Gal xenoantigen on glycolipids and third, the deletion of Galα1,3-Gal epitope that would expose new, potential, immunogenic antigens, whose role is currently overshadowed by the preponderant reactions of the α-Gal (Gock et al. 2011). In the human organism, even 90 % xenoreactive antibodies recognize Galα(1,3)Gal epitope, but there are still 10 % to be challenged. The second well-known xenoantigen is *N*-glycolylneuraminic acid. Neu5Gc is the common mammalian sialic acid that is not synthesized in humans who are genetically defective in production, but can metabolically incorporate it from dietary sources, maintaining high levels of circulating anti-Neu5Gc antibodies at the same time. Immune recognition studies by Lutz et al. (2013) showed that pigs lacking both CMAH (cytidine monophosphate-N-acetylneuraminic acid hydroxylase) and GGTA1 gene activities reduced the humoral barrier to xenotransplantation, further than pigs lacking only GGTA1.

Chen et al. (2005) using GTKO porcine endothelial cells unmasked subsequent non-Gal antigen. This protein of 47 kDa may be one of the major antigens that induce non-Gal antibody production during rejection. The magnitude of the response of induced antibodies to non-Gal antigens, including complement-dependent cytotoxicity to GTKO lymphocytes or endothelial cells, was well associated with severity of rejection, suggesting that induced antibodies against non-Gal epitopes were responsible for acute humoral xenograft rejection.

As a consequence of the complexity of the processes, we chose two strategies involving molecular modifications of the animal genomes aiming at preventing the HAR from the immunological system. First, research of our team concerning the design of transgenic pigs for xenotransplantation purposes includes genetic modifications of donor’s genome leading to a decrease in the α-Gal epitope level on the surface of cells and prevention of hyperacute reaction of the human immunological system by multiplication of the number of α1,2-fucosyltransferase copies. This allows for a modification of the oligosaccharide structure in donor cell surface proteins by the formation of a neutral H structure and reduction of the α-Gal antigen amount, which translates into a decrease in immunogenicity. Costa et al. (2008) demonstrated that chondrocytes from a transgenic pig with an expression of a gene coding for α1,2-fucosyltransferase display a twofold decrease in the sedimentation of the complement system elements, C3 and C4, as compared with the control, and a decreased amount of the α-Gal epitope. The results suggest that α1,2-fucosyltransferase expression in porcine cells may protect pigs from humoral or cellular rejection. This is confirmed by the results of our studies, which showed a significant reduction in α-Gal antigen on the surface of cells in transgenic animals with additional copies of α1,2-fucosyltransferase introduced to their genomes, compared with the amount of the xenogeneic epitope on the surface of cells in non-transgenic animals (Lipiński et al. 2010). α1,2-Fucosyltransferase located within the medial compartment of the Golgi apparatus cisterns shows its activity earlier than the *trans* endogenous α1,3-galactosyltransferase present in the trans compartment. The maturing oligosacharide, when migrating through the Golgi apparatus from the *cis* to *trans* compartments, cannot accept the terminal galactose in a reaction catalyzed by α1,3GT because of previous fucosylation of *N*-acetyllactosamine resulting from α1,2FT activity (Hartel-Schenk et al. 1991). Simultaneous transformation of COS cells with α1,2FT and α1,3GT coding genes showed the shift of the expression profile towards the production of the H epitope (Sandrin et al. 1995).

The complexity of processes responsible for initiating immunological reaction does not allow, however, for a one-dimentional approach to xenotransplantations. Several strategies need to be combined to obtain an additive effect in one transgenic organism, which increases the chance of inhibiting the immunological aggression against the cells of a xenotransplant and, as a result, its survival. Since the expression of α1,2FT does not result in total Galα(1,3)Gal epitope elimination, introducing additional copies of the gene coding for the enzyme, which would supplement the activity of 1,2-fucosyltransferase, seems necessary. Introducing a gene coding for α-galactosidase which removes the terminal α-d-galactose should result in a reduction of the functional Galα(1,3)Gal epitope formed despite the competition between α1,3GT and α1,2FT. Thus, it seems justifiable to use a strategy which aims to obtain double transgenic animals with α1,2-fucosyltransferase and α-galactosidase expression. Cytometric and cytotoxic analysis of cells from pigs with the α-galactosidase gene conducted by Zeyland et al. (2013) revealed a considerable reduction of the α-Gal epitope on the surface. Comparative analysis conducted for a line of fibroblasts derived from a transgenic animal with α-galactosidase expression revealed a minor decrease of cell survival rate in the test medium containing human complement system elements (91.67 %, ± 3.13 %) as compared with the basic medium, which did not contain those elements (98.53 %, ± 0.06 %) (Zeyland et al. 2013). Research by Jia et al. (2004) implementing both the strategies confirmed that the activity of α-galactosidase decreased the α-Gal epitope level by 78 % and the co-expression of α-galactosidase and α1,2-fucosyltransferase eliminated it entirely from the surface of cells (PEDSV.15).

Monotransgenic animals obtained as a result of the work of the team were used for the following interbreeding: an individual with the expression of a transgene containing a sequence coding for α-galactosidase was cross-bred with an individual with the expression of a transgene containing a sequence coding for α1,2-fucosyltransferase. The interbreeding resulted in 12 living piglets, including four individuals with confirmed integration of the pCMVFUT gene construct and three transgenic individuals with confirmed integration of the pGAL-GFPBsd gene construct, including one with a confirmed integration of both the gene constructs. The obtained results of the molecular and cytogenetic analyses as well as the analysis of expression in the double transgenic individual showed a stable integration and functionality of both the transgenes (Figs. 1, 2, 5).

The use of PCR screening allowed the identification of potential transgenic animals and reduced the number of samples for Southern analysis. From screening by Southern analysis, we were able to determine that transgenes were present, intact, in how many copies and what orientation (Tinkle and Jay 2002). The approximate number of pGAL-GFPBsd transgene copies integrated was estimated as 16 and the approximate number of pCMVFUT transgene copies integrated was estimated as 3 (Fig. 4). The obtained results are consistent with the results of our previous experiments concerning the founders of pGAL-GFPBsd family and pCMVFUT family (Lipiński et al. 2010; Zeyland et al. 2013).

Chromosome stability and incorporation of α1,2-fucosyltransferase and α-galactosidase transgenes in the founders’ genome into the active transcription regions resulted in their correct functioning in a descending double transgenic individual TG632 (Fig. 5). This fact shows that interbreeding of monotransgenic individuals proved to be an efficient and effective method of obtaining politransgenic animals. Two different modifications in one animal allow for a more efficient attempt to decrease the risk of transplant rejection. As demonstrated by the author’s own research, in single transgenic animals with α1,2-fucosyltransferase or α-galactosidase expression, the reduction of the α-Gal epitope is not full, which necessitates a simultaneous activity of two enzymes, α1,2-fucosyltransferase and α-galactosidase, in one organism. The conducted cytometric test on the reduction of the α-Gal antigen on the cell surface of fibroblasts from the three primary cell lines derived from an individual with α1,2-fucosyltransferase expression, individual with α-galactosidase expression and individual with α1,2-fucosyltransferase and α-galactosidase expression demonstrate the most efficient (threefold) reduction in the double-transgenic animal as compared with the fibroblasts in the non-transgenic pigs. It is worth remembering that BS-IB4 lectin isolated from *G. simplicifolia* used in flow cytometry investigation is not an antibody. Its reactive group is able to specifically interact with the carbohydrate components, without excluding affinity for similar groups even if devoid of immunogenic reactivity. Thus, recognition is not fully specific (Naso et al. 2012). In terms of specificity of α-Gal reactive site recognition, the comparison between lectin-based and the monoclonal antibody M86-based procedures before further clinical experiments will be needed. Regardless of the results of flow cytometry, cytotoxicity assay clearly demonstrated the functionality of introduced transgenes. Tests on cytotoxicity of human serum complement system elements in three individuals: with α1,2-fucosyltransferase expression, α-galactosidase expression and α1,2-fucosyltransferase and α-galactosidase expression revealed a decrease of cell survival rate in the test medium (containing human complement system elements) by approx. 6.57, 8.67 and 4.35 % in the 3 animals, respectively, as compared with the basic medium, which did not contain those elements.

Osman et al. (1997) reported that there was a greater reduction in surface Galα(1,3)Gal achieved by using a double transfection strategy with α-galactosidase and α1,2-fucosyltransferase than with either of the enzymes alone. IB4 staining was not detected on COS cells expressing both α-galactosidase and α1,2-fucosyltransferase, whereas IB4 staining of cells expressing only α-galactosidase was reduced by ≈ 50 % and of cells expressing only α1,2-fucosyltransferase was decreased ≈ 90 %. The additive effect of these two enzymes was reflected in the susceptibility of these cells to complement-mediated lysis in the presence of natural human serum with a maximal reduction in lysis to background levels when both enzymes were expressed in transfected cells. Unfortunately, expression evaluation tests within this research study did not reveal any additive effect of the activity of both the enzymes as compared with the individual with pCMVFUT expression and individual with pGAL-GFPBsd expression. Lack of cumulative effect of the co-expression of both the transgenes was also observed by other research teams (Houdebine 2002). In our case, this lack of a clear accumulation effect with regard to the activity of both the enzymes might stem from the fact that the cells we analyzed were heterozygous for both the transgenes. In order to verify the cumulative effect, research should be continued in homozygous cells.

In parallel with molecular studies, clinical trials are ongoing. As a part of our xenotransplantation project, several independent medical teams are working on the use of heart valves, bone and skin grafts of transgenic pigs for saving human health and life. Clinical research shows that the skin of genetically modified pigs can successfully be used as a biological dressing for patients with severe body burns and chronic wounds. Furthermore, based on histopathological studies it was found that the healing of the human bone grafts transplanted into transgenic pigs runs with less inflammatory reaction comparing with nontransgenic animals (data not published).

In conclusion, our research fits the global trend of designing politransgenic pigs, whose characteristics make it possible to overcome the multi-level barriers in xenotransplantation. The production of transgenic animals is a combined work of an interdisciplinary team of experts in molecular biology, reproduction biology, biotechnology, virusology and surgery. The list of organs and tissues that could successfully be transplanted into the human organism is extensive and includes heart, kidneys, liver, lungs, pancreas and skin (Cooper et al. 2012; Ekser et al. 2009; Hering and Walawalkar 2009; Le Bas-Bernardet et al. 2011; McGregor et al. 2012). Researchers face the challenge of developing the most effective combination of genetic modification in the donor organism. Their success would allow for a transfer of the technologies to the domain of human transplantology (Cooper et al. 2012; McGregor et al. 2012; Puga Yung et al. 2012).
